# Nutritional Modulation, Gut, and Omics Crosstalk in Ruminants

**DOI:** 10.3390/ani12080997

**Published:** 2022-04-12

**Authors:** Mohamed Abdelrahman, Wei Wang, Aftab Shaukat, Muhammad Fakhar-e-Alam Kulyar, Haimiao Lv, Adili Abulaiti, Zhiqiu Yao, Muhammad Jamil Ahmad, Aixin Liang, Liguo Yang

**Affiliations:** 1 Key Lab of Agricultural Animal Genetics, Breeding and Reproduction of Ministry of Education, Huazhong Agriculture University, Wuhan 430070, China; mohamed.asad@agr.au.edu.eg (M.A.); w18256163137@163.com (W.W.); aftabshaukat40@gmail.com (A.S.); miaomiaoxiyuhuai@hotmail.com (H.L.); adiliabulaiti@webmail.hzau.edu.cn (A.A.); zhiqiuyao1@163.com (Z.Y.); jameel_uaf@webmail.hzau.edu.cn (M.J.A.); lax.pipi@mail.hzau.edu.cn (A.L.); 2 Animal Production Department, Faculty of Agriculture, Assuit University, Asyut 71515, Egypt; 3 College of Veterinary Medicine, Huazhong Agricultural University, Wuhan 430070, China; fakharealam786@hotmail.com; 4 National Center for International Research on Animal Genetics, Breeding and Reproduction (NCIRAGBR), Huazhong Agricultural University, Wuhan 430070, China

**Keywords:** feedomics, gene expression, nutrigenomics, nutrition, transcriptome, ruminant

## Abstract

**Simple Summary:**

Over the last decade, animal nutrition science has been significantly developed, supported by the great advancements in molecular technologies. For scientists, the present "feedomics and nutrigenomics" era continues to evolve and shape how research is designed, performed, and understood. The new omics interpretations have established a new point of view for the nutrition–gene interaction, integrating more comprehensive findings from animal physiology, molecular genetics, and biochemistry. In the ruminant model, this modern approach addresses rumen microbes as a critical intermediate that can deepen the studies of diet–gut interaction with host genomics. The present review discusses nutrigenomics’ and feedomics’ potential contribution to diminishing the knowledge gap about the DNA cellular activities of different nutrients. It also presents how nutritional management can influence the epigenetic pathway, considering the production type, life stage, and species for more sustainable ruminant nutrition strategies.

**Abstract:**

Ruminant nutrition has significantly revolutionized a new and prodigious molecular approach in livestock sciences over the last decade. Wide-spectrum advances in DNA and RNA technologies and analysis have produced a wealth of data that have shifted the research threshold scheme to a more affluent level. Recently, the published literature has pointed out the nutrient roles in different cellular genomic alterations among different ruminant species, besides the interactions with other factors, such as age, type, and breed. Additionally, it has addressed rumen microbes within the gut health and productivity context, which has made interpreting homogenous evidence more complicated. As a more systematic approach, nutrigenomics can identify how genomics interacts with nutrition and other variables linked to animal performance. Such findings should contribute to crystallizing powerful interpretations correlating feeding management with ruminant production and health through genomics. This review will present a road-mapping discussion of promising trends in ruminant nutrigenomics as a reference for phenotype expression through multi-level omics changes.

## 1. Introduction

Ruminants are distinctive, influential animal species that have become worthy of attention in human food security marathons. The global population is expected to approach 9.15 billion people by the year 2050 [1], and in turn, global food animal production is expected to rise 2.3% annually, which will require rising production proportions [1,2]. Additionally, rapid population growth has made the animal production situation more critical [3,4], besides the presence of severe environmental changes (climate change, global warming, methane, and greenhouse gas emissions) and natural resource limitations (drought and desertification) [5,6,7,8]. Such emerging threats put pressure on the global situation of the animal protein supply due to the feed resource competition with human food production, which disrupts the sustainability of livestock production systems [9].

The classic animal nutrition approach was traditionally dominated by direct studies that examined the feeding practices related to the production phenotypes. However, this approach could not provide enough knowledge about the nutrient dynamics in the GIT, its effect at the tissue level, and, in turn, its reflection in the animal’s productivity. Additionally, it could not explain the mechanism of action of intermediate metabolites in different cellular activities through different tissue types.

Hence, the advancements in molecular biology, molecular nutrition and physiology, high-throughput technologies, and bioinformatics databases have led to the more powerful inclusion of other studies such as epigenetics, metagenomics, metabolomics, transcriptomics, and proteomics. However, these integration trends focused on the diet’s characteristics, its role in altering metabolism, and its effects on the pathways of other metabolites [10,11]. Thus, debates continue about the best strategies for epigenetic interference applications for determining more precise animal requirements that can guide genetic selection programs. Additionally, many questions have been raised about innovative approaches that broaden our interpretations for more efficient feed resource utilization.

“Omics” refers to methodologies that relate to the knowledge about specific identifiable genes in an animal or the microbiome, genes transcribed to mRNA or proteins, and metabolites present within a particular cell, tissue, organ, fluid, or population [12]. Some of the published literature has drawn attention from traditional nutrition studies toward a closer look at feedomics and nutrigenomics. Feedomics is a field of study that looks at how changes in the diet and gut can affect gene expression, and it is also proposed as the “feed-gut-gene scheme.” In comparison, “nutrigenomics” focuses on nutrient molecules’ role in gene expression and the regulatory mechanisms generally at the cellular level [13].

Recently, feedomics and nutrigenomics have made their way to precisely illustrate the nutritional interventions in animal genetics which can open space for genotypic-tailored feeding studies. This revolutionary approach has focused on how feeds talk to genes and how genes respond, addressing a novel holistic approach and redefining the conventional ruminant nutrition–gene pattern in a broad context. Moreover, animal bioscientists have highlighted the host rumen milieu as a critical intermediate player that controls, regulates, or triggers serial changes by the rumen microbiome’s activities [14,15].

This review discusses feeding and nutrition strategies from a molecular genomics point of view. It introduces a larger framework that places the feed–gut context as the first step toward efficient ruminant nutrition for improving animal health and welfare.

## 2. Molecular Nutrition–Genomic Interferences

It has been reported that genes alone do not necessarily produce phenotypic traits; various environmental aspects can affect the incidence and degree of trait expression. Nutrition is a principal environmental factor; however, it needs profound genomic enlightenment due to the complexity of feeding-related phenotypes such as feed efficiency [16]. Additionally, our knowledge of which nutritional substrates may impact gene expression is limited. Further, the existing literature analyzes the whole scenario from the diet through the rumen to genes, although the final product is not fully discussed and remains inadequate.

Recently, studies have been published that group genomic feedback with ruminant feeding management and feed formulation. As a result, they have helped to determine more precise nutrient requirements for more sustainable strategies in ruminant production systems. Therefore, trials to understand the genetic response to nutrition have been further complicated and have provided an opportunity for novel research studies that can thoroughly explain the intricate relationship between diet and animal tissue genomics [17,18,19,20,21,22,23,24].

DNA microarrays and gene analysis applications could not prove RNA dynamics, whether mRNA synthesis (transcription) or RNA degradation. Therefore, the preference for RNA-based techniques is attributable to DNA’s existence in both active and inactive or dead cells [25]. However, RNA is dynamically distinguished in participating cells, making RNA a more accurate cellular biomarker. Therefore, RNA-based systems are more precise in omics studies, particularly microbial metabolic activity interpretations [26,27,28,29].

## 3. The Metabolism Messengers for Gene Regulation

In ruminants, researchers’ main challenge is investigating the relationship between metabolism and genes, tracking molecular pathways that primarily depend on an mRNA transcript methodology [28,30,31]. However, the link between mRNA abundance in the tissue and phenotypic or protein changes in tissue’s gene transcription is not simple because the regulatory pathway for protein synthesis is a multi-stage journey [30,31,32]. Previous studies have established the importance of investigating transcripts depending on the output protein’s significance in regulating or controlling specific metabolic processes [23,24,25,26,27,28,29,30,31,32,33,34,35,36]. Similar works also argued the effect of nutrition on proteomic changes and the feasibility of inducing them in ruminants, which are still scant and surpass the application of these studies to rodent models [37].

**Figure 1:**
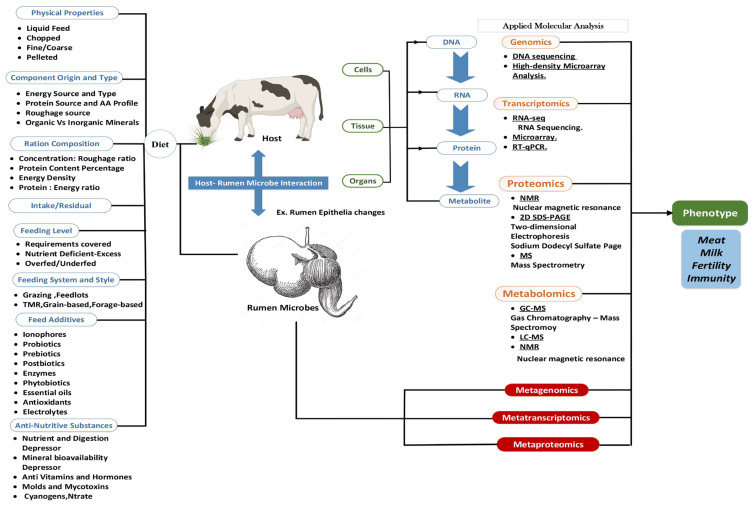
Potential of different dietary components and characteristics of molecular changes in a ruminant model.

As we will discuss later, studies in the literature have reported the potential of some dietary components to affect the cellular metabolism and growth transactions differently through the omics context [38,39,40]. However, such explanations are still unsatisfactory because each dietary factor may have a multi-genomic fingerprint distinguishing some metabolic activities linked to gene expression regulators [41,42,43]. Figure 1 presents the feed characteristics and the potential induction of molecular changes.

## 4. Tracking the Change Cascade across Gastrointestinal Tissues

**Figure 2:**
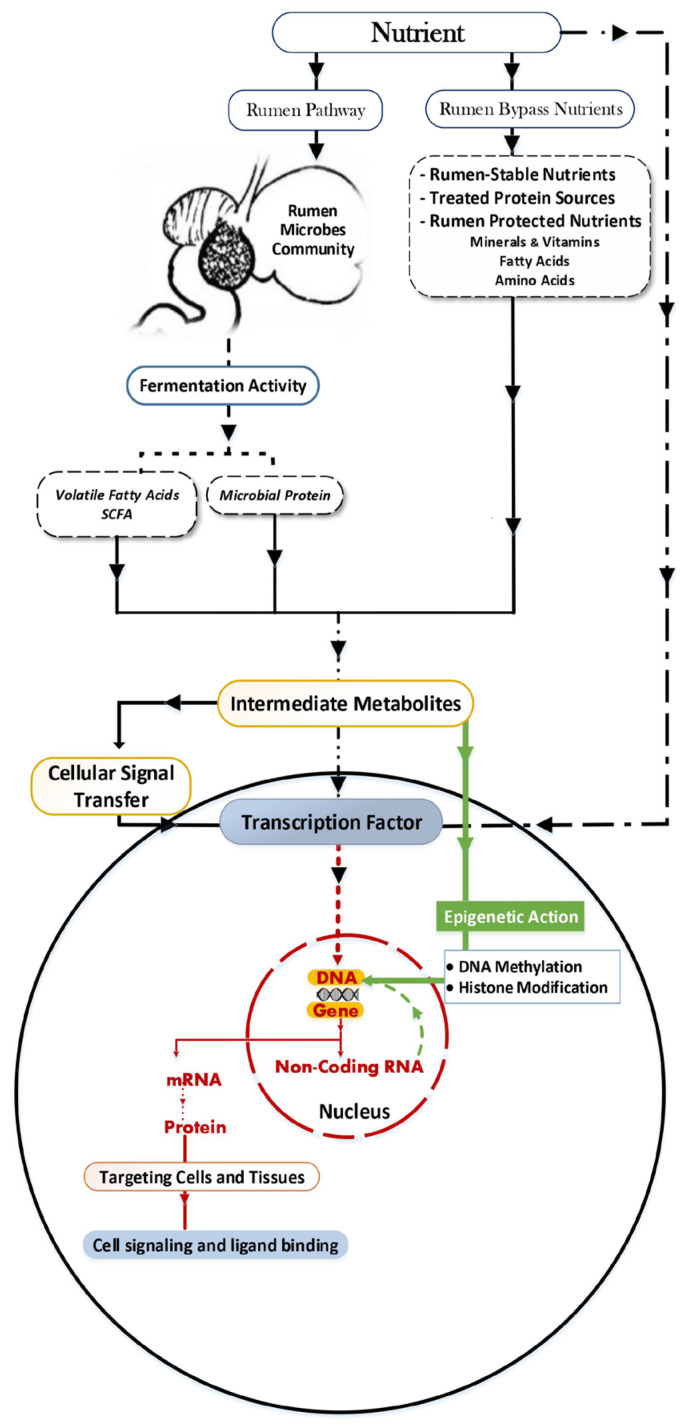
Nutrition–gene interaction pathway in ruminants.

Recent trends in feedomics have tracked changes at the feed level or the biochemical level such as an intermediate metabolite, mapping the pattern for multiple mRNA alterations [20,33,44,45,46]. Therefore, it was suggested that there are two paths for feed to start the molecular change cascade. Firstly, the GIT changes are induced by the feed’s physical or biochemical action on the rumen and intestinal tissue. Some physical changes such as papillae development affect absorption, post-absorption, and various metabolism functions [47]. For example, it was reported that 47.5 percent of the critical genes in the rumen epithelial tissues of beef steers are involved in metabolic processes [48]. Secondly, passing the baton to the volatile fatty acids (VFAs) results from the microbiota activity, which acts as a metabolic mediator. The VFA action mainly activates or depresses the specialized transcription factors (TFs) by binding to them (e.g., Figure 2).

### 4.1. Transcription Factors (TFs)

TFs are functional cellular proteins that manage the gene expression process through binding to target gene regulatory regions (silencer or promoter sequences) on the DNA, sparking gene expression series, and controlling the gene transcription rate [39]. Transcription factors are crucial but not the only mediators in the nutrient–gene scene. Recently, reports have shone the spotlight on the nutrient, mediator, and TF complex that is responsible for launching a later phase of gene upregulation [49]. The second wave of gene expression starts after the upregulation of subsequent TF transcription [49]. Previously, it has been reported that transcription factors may harmonically work in networks of transcription factors that respond to dietary factors [50].

There are various types of transcription factors such as peroxisome proliferator-activated receptors (PPARs), liver X receptors (LXRs), and retinoid X receptors (RXRs). The ligand-dependent nuclear receptors (LdNRs), such as PPARs (α, β, and γ), play a central role in the ruminant model [51]. PPARs are known for their vital cellular functions, such as fatty acid catabolism in skeletal muscle [52], regulation of glucose uptake [39], adipogenic actions [53], and fatty acid oxidation [54]. They are mainly activated by fatty acids, regardless of their source—either the diet or an intermediate metabolite as a ruminal fermentation product [34,38].

The LXR family has major regulatory functions for production traits, such as the two known isoforms α and β which are mainly activated by sterols and fatty acids [55]. For example, LXRα showed regulation capacity for SREBF1 (sterol regulatory element-binding transcription factor 1) expression, a crucial transcription factor regulating milk fat synthesis [55,56]. On the contrary, although it is known that retinoids (9-cis-retinoic acid) are the primary activator of RXR, there are limited data on the potential nutrigenomic effects of vitamin A and derivative retinoids such as 9-cis-retinoic acid through RXRα [57,58].

### 4.2. DNA Methylation

DNA methylation is a critical epigenetic mechanism that affects gene expression for parent-of-origin traits by methyl group addition without any DNA sequence change, affecting DNA activity. This process occurs by an enzyme group, “DNA methyltransferase (DNMT),” composed of five members: DNMT1, DNMT2, DNMT3A, DNMT3B, and DNMT3L. These enzymes’ mode of action includes methyl group addition to the fifth carbon of cytosine (C) in CpG dinucleotides, forming 5-methylcytosine. The methylation rate strictly correlates with the gene expression extent; hypermethylation of the promoter region depresses the expression level, whereas a low degree of methylation or hypomethylation refers to the active gene expression process [59,60]. Adaptation from parents to offspring is a big part of this mechanism, especially adaptation to various environmental conditions such as heat stress [61], a stimulus such as a change in maternal management [62], physiological state [63], mastitis [64], and milk protein synthesis [65].

### 4.3. Histone Modification

Histone modification is based on physical conformation to chromatin structure reform by adding or removing functional groups from the N-terminal tails of histone proteins such as H2A, H2B, H3, and H4 [66,67,68]. This conserved protein modification also includes lysine methylation, lysine acetylation, serine/threonine phosphorylation, and ubiquitination [69,70,71]. The majority of histone alterations can regulate the developmental style; the modification in the promoter region results in depressing or activating genes corresponding to different environmental stimuli, such as ultraviolet (UV) or other radiation and chemical carcinogens [72].

### 4.4. Non-Coding RNA (ncRNAs)

The majority of mammalian genomic DNA is transcribed as non-coding RNAs (ncRNAs) [73], which are initially defined as “junk” [74]. The main effects of ncRNAs range from interfering with mRNA stability to regulating mRNA transcription and translation [75,76]. In ruminants, researchers have shown an increased interest in long non-coding RNAs (lncRNA) and microRNAs (miRNAs), which are well-studied types of non-coding RNA.

#### 4.4.1. Long Non-Coding RNAs (LncRNAs)

LncRNAs refer to RNA transcripts greater than 200 base pairs possessing no protein-coding activity. They have been recently appreciated in physiological processes [77]. However, their examination among ruminants is still limited [27,28,78]. Although the precise acts of lncRNAs are not explicit yet, lncRNAs are reported to have a potential regulatory function in the bovine mammary gland through the pathway of lipid metabolism, fatty acid synthesis [78], and calves’ intestinal growth [27].

#### 4.4.2. MicroRNAs (miRNAs)

MicroRNAs (miRNAs) are non-coding RNAs (18–25 nucleotides) that play an essential role in many physiological processes. Moreover, it is estimated that miRNAs form between 1% and 5% of animal genes and are expected to control at least 60% of genes involved in all cellular activities [79]. The interesting role of miRNAs presents through regulating RNA readiness in the posttranscriptional phase and before translation, affecting protein derivation [80,81,82,83,84]. Additionally, miRNAs are well known for their various significant biological functions, such as adipose tissue regulation [85], proliferation and differentiation of gastrointestinal tissue cells [45,82,86,87,88], mammary gland development [26,33,89,90,91,92], and ovary development [85,93]. Therefore, studying the expression and distribution of miRNAs has attracted interest across a wide range of tissues, aiming to interpret diverse cellular mechanisms, particularly from a pathological perspective [94].

Furthermore, the expression and function of specific miRNAs can be modulated by nutrition. For example, in lactating goats, the expression of 30 miRNAs in the mammary gland was modulated through macronutrient deprivation, where 14 miRNAs were upregulated, and 16 miRNAs were downregulated [53].

Thus, the animal gut’s tracking of an inherited genetic change might take different forms depending on the nutrient or the nature of the feed, shaping gene expression, DNA, and histone modification.

## 5. Nutrition Influence in Tracking of Epigenetics

**Figure 3:**
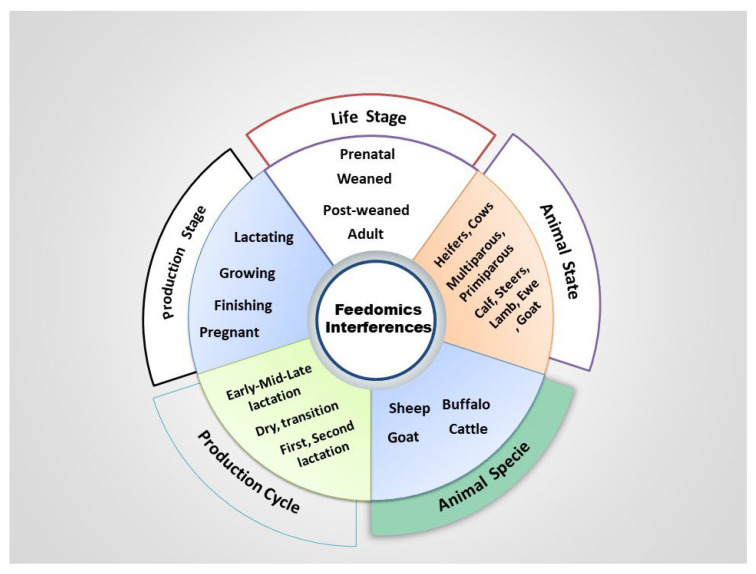
Animal factors that can change the feedomics imprint.

Among environmental factors, nutrition can induce desirable epigenetic effects [95,96,97,98] for some traits such as fertility [99,100]. However, diet–epigenetic intervention, or the linkage between nutrients and inheritable changes in DNA base pairs, primarily occurs through chemical regulation mechanisms. As depicted in Figure 3, the potential interaction between environmental conditions and animal status can alter the epigenetic style. Furthermore, broad findings have focused on dietary components and various metabolites as signal messengers for cellular activity in reproductive tissues and organs, as well as their significant effect on reproductive efficiency [101,102,103,104,105]. Additionally, fertility–epigenetic studies supported comprehensive nutritional management as an applicable tracking tool for potential reproduction improvements. In addition, it was reported that fatty acids, especially polyunsaturated types, can alter reproductive performance during different life stages, which is also linked to adipose tissue gene expression [106,107,108,109,110,111,112,113,114].

Conversely, some nutrient substrates may have a contradictory action, even though VFAs play multiple roles across several physiological activities as energy sources and significant transcription factor agonists [39]. Nevertheless, they have been shown to inhibit histone deacetylase mechanisms [115]. Epigenetically, some modifications to DNA base pairs do not change the DNA sequence itself but can shape transgenerational transcription phenotypes (e.g., Table 1).

## 6. Fetal Programming in the Nutrigenomics Context

Maternal nutritional management has a significant impact, especially in late pregnancy when colostrum is secreted: for instance, selenium supplementation and raised IgG levels in the cattle colostrum [125]. Additionally, in pregnant sheep, changing hay-based diets to corn-based diets in the second half of gestation significantly depressed the expression of (H19, MEG8, PEG1, DLK1, and IGF2R) DNMT genes in the fetus muscles. The expression of these genes was found to be associated with embryonic programming and muscle growth [126].

Moreover, pregnant ewes supplemented with protected methionine in late gestation produced lambs heavier than those produced by non-supplemented ewes [127]. Similarly, treating dairy cows with dietary protected methionine in the late gestation upregulated placental genes that participate in neutral AA and glucose transport, accompanied by higher gene and protein expression of mTOR; this change was also associated with increased calf birth weight [128]. Although some studies have reported that maternal nutrition during late gestation could be a way to change the offspring’s miRNA in beef cattle, there are still some questions about the correlations between colostrum’s miRNAs and their effect on offspring [129,130]. Maternal nutrition substantially affects offspring signaling pathways via regulating transplacental transfers [131] or other diverse pathways [118,119,130,131]. Available nutrients mainly pass through a channel in “the placenta” for launching pathways of signaling such as controlling amino acid transport, as is the case for the mammalian target of rapamycin (mTOR) complex, or the peroxisome proliferator-activated receptor γ (PPARγ), which is the leading influencer of lipid pathway regulation.

## 7. Nutrigenomics during Newborn Animal’s Life

Although the placenta serves as the primary fetus transfer channel for nutrients and other signaling molecules that pass from mother to newborn, colostrum is thought to be the first super source of active proteins, minerals, and vitamins. However, the placenta barely delivers some bioactive molecules such as immunoglobulins, the chief molecules that can hardly be transferred through it [132]. Therefore, colostrum is the sole source of immunoglobulins that play a crucial part in an animal’s lifespan and passive immunity. Previous studies highlighted growth promoters in colostrum such as insulin-like growth factor (IGF-1) and hormones, focusing on colostrum management and its effects on gut development in neonatal animals [133,134]. In the first week after birth, ingesting colostrum could regulate the expression of T and B cell lineage-specific genes in the intestinal mucosa, in addition to miRNAs and microbial colonization, which may control various mucosal immune changes [135]. While delaying the first colostrum administration after birth reduces IgG transfer in calves [136], calves that ingested colostrum had a higher serum content of amino acids (leucine, valine, and glutamate), which are known for their health benefits and immune expression induction, particularly in the colonic mucosal immune system [137,138].

Among the various colostrum components, the higher content of miRNAs becomes an interesting feature that distinguishes this newborn liquid feed from mature milk that can pass through the milk in bovines [139]. Similarly, miRNA is notable for its immune participation effect on B and T cell differentiation, and interleukin production of macrophages [140]. However, colostrum’s miRNAs drew attention as an active biological component and were remarkably nominated as signaling molecules communicating between the mother and her offspring [141]. Many dairy performance fingerprints mentioned that bta-miR-574, which regulates the leptin receptor, controls the development and lactation of mammary tissue in dairy goats [33]. During lactation, the maternal dietary fat content is suggested to be a fundamental controller of miRNAs in colostrum [141]. However, miRNAs related to lipid metabolism may not be associated with changes in energy sources [142].

The above description is not the whole story of nutrigenomics; studies could not fully discover a central role player—a rumen microbe—which contributes through meta-transcriptomic or meta-proteomic factors. Thus, the rumen microbes may be the critical responders for nutritional change and thus launch another wave of serial changes as we are about to discuss.

The neonatal gut microbial community is a strategic partner in calf health and performance. Since microbial colonization starts from the first days of neonatal animal life, it interacts actively with the first diet in early animal life. Therefore, significant rumen development changes can be found in an age-dependent manner [143]. Additionally, through the microbe–host context, reports have proposed that rumen microbiome changes could regulate neonatal gut development [144]. Dietary promotion of a diverse microbial community is mainly favored as an infection-preventive measure in this sensitive stage. It promotes beneficial bacteria colonization in the small intestine, constraining pathogen microbes’ colonization [145,146]. Newborn calves with a lower incidence of diarrhea and higher growth rates tend to have a higher fecal prevalence of Faecalibacterium, a butyrate-producing strain, and major acetate consumers, which intensify the energy content per mole of the ruminal VFAs. Noteworthy, it plays a partial anti-inflammatory role in Faecalibacterium prausnitzii due to the production of metabolites that further depress NF-kB activity and IL-8 production [147].

## 8. Feedomics and Nutrigenomics Strategies through Premature Diets

Pre-weaned feeding depends on colostrum, milk replacers, or even whole milk, which passes directly to the abomasum due to the esophageal groove’s existence. As a result, newborn ruminants’ reliance on liquid feeds may limit rumen development [18,148,149]. Furthermore, in the rumen epithelium, MCT1 is the major cellular monocarboxylate transporter (such as SCFAs, lactate, pyruvate, and ketone bodies). MCT1 is mainly responsible for transferring energy sources from the ruminal epithelial cells to the bloodstream and maintaining the intracellular pH [148]. Therefore, MCT1 expression in neonatal ruminants may be influenced by liquid feeds [149], intraluminal SCFA concentrations, or a lower pH value [150]. In beef-producing calves, it was found that adopting a strategy of early weaning (at two months of age) and introducing different diets (high dietary starch) resulted in precocious adiposeness activity present in more intramuscular fat deposition, producing higher-graded carcasses. These dynamics in skeletal muscle tissue activated by the dietary change are mainly coordinated by PPARγ and CCAAT enhancer-binding protein alpha (CEBPA) [151].

Various dietary alterations in premature animals can affect their upcoming production patterns through genomic alteration. For example, starter enrichment (especially for protein content) in neonatal Holstein calves elevated PPARA and cell proliferation gene expression (INSR, FOXO1, AKT3) [152]. Additionally, this change was accompanied by upregulated ketogenic genes (HMGCS2, HMGCL, and BDH1) simultaneous to fatty acid oxidation gene (CPT1A, ACADVL) downregulation, mainly suggesting that early dietary enhancements may be a promising route for promoting energy utilization in the ruminal cell, which results in more significant ruminal development [152]. In addition, changes in the early feeding strategy and style of newborn ruminants may influence rumen development and initiate long-term consequences for lifetime productivity [141,142,143,144,145,146,147,148,149,150,151,152,153,154,155,156].

## 9. Feed Efficiency and Gene Expression

Productive, healthy animals require an adequate intake of tallied and well-balanced diets. Caloric density and nutrient availability are among the controllers of metabolism through gene expression by inducing changes in metabolic regulatory signals, mainly since nutrient supply and hormonal status are strictly linked [103,157]. Moreover, low-feed-intake animals are more vulnerable to several immune responses such as inflammation, liver lesions, and bacterial infection [158]. Additionally, efficient animals are the valuable producer’s target because, economically, this means less feed consumption and lower production costs. Moreover, efficient animals showed further environmental benefits such as lower ammonia emissions [159], 28% less methane [160], and 15% less manure [161].

Feed intake and residual feed intake (RFI) have been used as expressions for feed efficiency measures. However, RFI is calculated as the difference between actual feed intake and estimated feed intake on a maintenance and growth requirement basis. Low-RFI animals are considered efficient, whereas high-RFI animals are considered inefficient. Since it is based on energy intake and requirements contrary to the gain: feed ratio, RFI is unrestricted by growth outlines, making RFI a more reliable feed efficiency measure. Researchers highlighted RFI as a precursor to animal energy intake. This opened possibilities to apply genomic selection to this trait, a moderately heritable trait (0.28 to 0.45), to identify genes associated with various physiological pathways [162,163]. Correspondingly, feed efficiency measures were integrated with selecting feed-efficient animals in time-saving, accurate, and cost-efficient styles [158]. Suggested regulatory genes for energy production linked to RFI were also associated with paracellular permeability, which assists various nutrients’ and SCFAs’ transport [88,148]. In beef cattle, low-RFI animals showed higher expression for a group of genes (TPI1, TECR, COX8A, SLC25A39, PKM2, and SUZ12) that play a part in rumen epithelium morphogenesis through facilitating energy production, needed for tissue development [48].

As feed intake changes, the ruminant GIT reacts differently to pH disruption. The ruminal epithelium responds in various ways; as a short-run response, the molecular adaptation includes greater gene expression and proteins participating in VFA transport actions [162,163]. Therefore, the Na^+^/H^+^ exchanger’s activity (such as SLC9A1) tends to show elevated expression, which has been nominated as adaptive molecular-physiological feedback for stabilizing pH through the rumen and omasal epithelium [164,165], and higher expression of Na^+^/H^+^ exchangers linked to insulin signaling [166]. Then, physical adaptation follows, through expanding absorptive surfaces by the morphological development of the ruminal epithelium, such as hyperplasia and hypertrophy [10,167]. Some of the discussions provided a molecular understanding of the ruminal epithelial absorptive mechanism in feed-efficient animals. VFA uptake synchronized with absorption and upregulating genes in the ruminal epithelium [168,169]. Upregulation of VFA absorption enhances VFA uptake in ruminal epithelial cells, which results in an increased pH level through an elevation in intracellular hydrogen ions to normalize the intracellular pH status [170,171].

## 10. Genomic Changes through Dietary Management

Feed restriction protocols have frequently been used to examine intake reduction’s effect, its relation to mRNA abundance in GIT tissues, and potential feeding behavior feedback. Previous studies have shown that feed restriction could downregulate specific gene expressions such as α-lactalbumin (LALBA), which is mainly considered responsible for expressing co-enzymes that participate in lactose synthesis, which explains the milk production decline for restricted feed cows [172]. However, it was reported that during short-term feed deprivation, GIT hormones’ (cholecystokinin and glucagon-like peptide 1) concentration decreased due to mRNA abundance depression of these hormones in the duodenum and ileum [37].

Several studies have shown that dietary energy might play an essential role in how different tissues use other nutrients. For example, dietary energy and propionate production could help bovine mammary tissue make more protein [38]. In addition, previous studies have shown that dietary fatty acids can change cellular behavior. For example, they can change the miRNA regulation of ovine adipogenic genes [173] or make a specific gene more active, which might be an inflammatory mediator such as L-selectin [174]. Furthermore, controlled energy intake also confers ruminant advantages by triggering hepatic molecular adaptations well ahead of parturition [175]. In this connection, intensifying the dietary caloric content using unsaturated fats is more favorable than using oils. This preference for saturated over unsaturated fats in ruminant diets is due to the higher digestibility of saturated than unsaturated forms, which also depress milk fat [176]. It is noteworthy that the abundance of mRNA transcripts in pregnant, repeat-breeding cows that were fed n-3 PUFA-rich diets showed upregulated interferon-stimulated gene (ISG) expression, accompanied by an increased preovulatory follicle (POF) size [21]. Additionally, n-3 PUFA supplementation was correlated with suppressing the pulsatile endometrium secretion of PGF2α that had anti-luteolytic activity [109], besides higher embryonic survival [177].

In the same vein, energy overfeeding of dairy cattle in the dry period has been linked to transcriptional changes, disposing cows to fatty liver, and perhaps overall liver health during the periparturient period [175]. Moreover, it has been conclusively shown that higher-feed-intake beef steers showed significant increases in gene expression responsible for cell growth and proliferation, highlighting factors associated with glycolysis and oxidative phosphorylation in rumen epithelial cells [48].

It is thought that cutting back on food could affect reproductive traits and growth in the small ruminant model. In addition to gut morphology impairments, early feed-restricted ewe lambs showed inefficient reproduction performance and retarded live body weights [178]. Contrarily, this suggestion raises the negative consequence of the acidic effects of high-energy diets that depress cell barrier capacity against various damaging molecules. In addition, an energy-rich diet could weaken some rumen epithelial cellular immunity functions by depressing the expression of some proteins such as HSP71 [18].

Furthermore, various proteins’ abundance and shifts in the genes expressed in the ruminal epithelium showed a linkage to metabolite flux. This abundance, which may be related to changes in ruminal bacterial species [179], was closely related to the metabolite profile, with significantly higher ruminal SCFA concentrations, particularly valerate and butyrate [180,181,182]. Moreover, butyrate, the key influencer in epithelial barrier integration [183], also affects the expression of genes contributing to other SCFA transports through the ruminal epithelium (SLC16A3, SLC26A3, and HIF1A) in sheep and (PAT1, AE2, MCT1, and NHE2) in goats [184,185]. Noteworthy, short-chain fatty acids (propionate specifically) could decelerate GH expression and prolactin (PRL) in dairy cow anterior pituitary cells [186].

## 11. The Future of Research in GIT Mucosal Immunity

The previous sections integrated the diet–GIT development of neonates with mature animals, linking it with transcriptomic changes. These things are essential to understanding how animal performance can be affected by changes in nutrition through the gut.

The previous results highlighted that the mucosal epithelial architecture change could result in antigen changes and various innate immune responses followed by a disturbance in cytokine profiles. Therefore, diet–microbiota and host immune modulation interventions against gastrointestinal pathogens can significantly optimize production performance and minimize gastrointestinal disease [187]. Additionally, such interventions are mainly responsible for early life stage stress [188], which significantly depresses the newborn animals’ growth performance and health. However, there is a knowledge gap about the mechanisms involved, especially from the host side of this host–diet interaction. Additionally, novel molecular approaches such as fecal microbiome RNA can enrich this research spot and introduce a more deep interpretation of the dynamics of the cellular changes during the different animal life stages [189].

Therefore, the mucosal immune functions have opened future questions about whether the GIT mucosal immunity can be a starting point for re-evaluating nutritional management and strategies, especially for ruminants.

## 12. Conclusions

Feedomics and nutrigenomics have revolutionized our previous knowledge about ruminant nutrition. The interaction between feeding and gene expression can be manipulated for more benefits concerning animal health, production sustainability, and welfare. DNA- and RNA-based technologies empower researchers to form a comprehensive picture of the feed effect on biological changes, and metabolic and epigenetic mechanisms. Additionally, feedomics and nutrigenomics studies revealed the critical role of the rumen microbiome that is present mightily in many physiological-metabolic pathways. Additional factors must be considered through feedomics studies, such as age, animal species, production phase, and gut–host relations. Different dietary diet/gene connections between production systems are complicated, especially with multi-gene expression changes. In all the studies reviewed here, nutrigenomics insights support researchers in remodeling feeding practices efficiently and isolating diet-induced changes from other causes of change such as age and development. It is hoped that this review will help to build a bigger picture that can show how each dietary component has a unique genomic response that can be used in future feeding management strategies.

## Figures and Tables

**Table 1:** Dietary characteristics and components that have an epigenetic effect.

Factor	Action	Animal Type	Reference
Maternal protein insufficiency	DNA methylation	Sheep	[116]
Vitamin b12, folate, and methionine deficiency	DNA methylation	Sheep	[117]
Rumen-protected	DNA methylation	Cows	[118]
methionine			
Maternal undernutrition	DNA methylation	Sheep	[119]
Maternal overnutrition	Sex-specific DNA methylation	Sheep	[120]
Methionine supply	Sex-specific DNA methylation	Cows	[121]
Undernutrition	MicroRNAs	Cows	[122,123]
	Histone modifications		
	DNA methylation		
Rumen-protected methionine	DNA methylation	Cows	[124]

## Data Availability

Not applicable.
